# Efficacy of one-surgeon basketing technique for stone extraction during flexible ureteroscopy for urolithiasis

**DOI:** 10.1080/2090598X.2021.1889943

**Published:** 2021-02-20

**Authors:** Go Anan, Kazunori Hattori, Shingo Hatakeyama, Chikara Ohyama, Makoto Sato

**Affiliations:** a Department of Urology, Tohoku Medical and Pharmaceutical University, Sendai, Japan; b Department of Urology, St. Luke’s International Hospital, Tokyo, Japan; c Department of Urology, Hirosaki University Graduate School of Medicine, Hirosaki, Japan

**Keywords:** Urolithiasis, ureteroscopy, lithotripsy, laser, solo surgeon

## Abstract

**Objective**: To evaluate the safety and efficacy of using one-surgeon basketing technique by a solo surgeon for stone extraction during flexible ureteroscopy (f-URS) for urolithiasis.

**Patients and methods**: This retrospective study enrolled patients with urinary calculus who underwent f-URS at two institutions in Japan between September 2014 and March 2020. A total of 100 patients were operated by one experienced surgeon using the one-surgeon basketing technique. With this approach, the f-URS apparatus was manipulated with the non-dominant hand and the basket catheter was manipulated with the dominant hand. We retrospectively examined the perioperative results, complications, and stone-free rate [with ‘stone free’ defined as ≤2 mm with kidney–ureter–bladder (KUB) at 1 month after f-URS] to estimate the safety and efficacy for comparison with the results of conventional retrieval basketing technique.

**Results**: Among our study population, the median stone size was 14 mm and median operative time was 74 min. A stone-free status was achieved in 91 patients (91%). The median stone fragmentation time was 15 min and stone retrieval time was 30 min. All included patients were treated using the one-surgeon basketing technique. Complications related to stone retrieval were identified in two patients (2%); the degree of ureteral injury was classified as Clavien–Dindo Grade IIIa.

**Conclusion**: The one-surgeon basketing technique is safe and effective for the extraction of stone fragments during f-URS for urolithiasis. This technique does not require assistance for basketing; therefore, f-URS with active retrieval basketing can be completed by a solo surgeon.

**Abbreviations**: BMI: body mass index; KUB: kidney–ureter–bladder; SFR: stone-free rate; UAS: ureteral access sheath; f-URS: flexible ureteroscopy

## Introduction

The treatment of urolithiasis has evolved over the years from open lithotomy to minimally invasive endoscopic lithotripsy [1]. In particular, the number of flexible ureteroscopy (f-URS) has dramatically increased alongside ongoing improvements in laser and surgical instruments [2,3]. Two basic options exist for the treatment of stones under URS. The basketing retrieval technique is used when stones have fragmented into pieces typically 2–4 mm in size, whereas the stone dusting technique is more appropriate when stones have fragmented to submillimetre pieces (mostly sized ≤2 mm). The latter technique is used to promote the spontaneous passage of stones through the ureter [3,4]. The potential advantages of the dusting technique over the basketing retrieval approach include a shorter operative time, reduced cost due to decreased use of a ureteral access sheath (UAS) and a basketing device, and decreased potential trauma associated with repeated basketing and UAS use [5–7]. Conversely, potential advantages of the basketing retrieval technique are a higher stone-free rate (SFR) and decreased postoperative stone-related pain and emergency visits postoperatively [5–7].

The process of stone retrieval using a basket device is a stressful task. It requires handling of a flexible ureteroscope while simultaneously manipulating the basket device. Thus, about two-thirds of urologists use the dusting technique that does not require a basket device during f-URS [3]. However, it has been reported that about 20% of cases who develop stone dust or small fragments of <4 mm experience emergency visits within 5 years following f-URS [8,9]. Basketing retrieval of stone fragments might help to alleviate such postoperative problems. Further, depending on the case, basketing retrieval is considered a necessary approach. However, there are few reports available regarding the use of the one-surgeon basketing technique [10,11]. In most cases, the two-person conventional extraction method has been used at the time of extraction during f-URS; therefore, the presence of a surgeon and an assistant is necessary during the surgery. In the present study, we evaluated the efficacy and safety of the one-surgeon basketing technique, which is a surgery performed by a solo surgeon. If the one-surgeon basketing technique is possible, even hospitals with a solo urologist could choose a retrieval method for f-URS. We consider this technique as one of the effective surgical methods for f-URS. The purpose of the present study was thus to evaluate the safety and efficacy of the one-surgeon basketing technique and discuss our experience with this technique.

## Patients and methods

This retrospective study included patients with urinary calculus who underwent f-URS from September 2014 to March 2020 at the St. Luke’s International Hospital and Tohoku Medical and Pharmaceutical University Hospital, Japan. The one-surgeon basketing method during f-URS was performed to extract stones. While more than 200 consecutive patients underwent f-URS during the above period, we included 100 patients performed by a single experienced surgeon (f-URS with one-surgeon basketing technique experience of >100 cases) using the one-surgeon basketing technique. Inclusion criteria included one-surgeon basketing method using a flexible ureteroscope. Exclusion criteria included: (i) stones sized >3.0 cm; (ii) complex stones that were initially scheduled for two-stage surgery; (iii) stone formation after urinary diversion; and (iv) use of only rigid URS in lithotripsy. This retrospective study was approved by the Ethics Committee of Tohoku Medical and Pharmaceutical University Hospital School of Medicine, Sendai, Japan (protocol 2019-2-056). Written informed consent was obtained from each patient.

### Equipment

The following equipment was used: 100-W holmium:yttrium aluminum garnet laser (VersaPulse PowerSuite; Lumenis, Yokneam, Israel) with a 200- or 365-μm laser fibre (SlimLine 200 or 365 μm; Lumenis), a flexible ureteroscope (7.5-F FlexX2; Karl Storz, Tuttlingen, Germany, or 7.95-F URF-P6; Olympus, Tokyo, Japan), a semi-rigid ureteroscope (6.0/7.5 F or 8.0/9.8 F; Wolf, Knittlingen, Germany), an irrigation system (single-action pumping system; Boston Scientific, Natick, MA, USA), a UAS (Flexor 12/14 F or 9.5/11.5 F; Cook Medical, Bloomington, IN, USA), a single-use basket holder (M-arm; MC Medical, Tokyo, Japan), and a basket catheter (N-gage, 1.7 F; Cook Medical).

### Surgical technique

All patients underwent a standard surgical procedure. In brief, a semi-rigid ureteroscope (6.0/7.5-F or 8.0/9.8-F rigid ureteroscope) was inserted into the urethra and the bladder was examined under general anaesthesia. A straight guidewire was inserted into the ureteral orifice on the diseased side, and the semi-rigid ureteroscope was passed over the guidewire through the ureteral orifice until safely reaching the renal pelvis region or ureteral stone. The presence or absence of ureteral stenosis or ureteral stones was confirmed using a rigid ureteroscope before UAS insertion in all patients. When there was ureteral stenosis, ureteral dilatation was performed or a ureteral stent was placed and two-stage f-URS procedure was scheduled.

**Figure 1.**
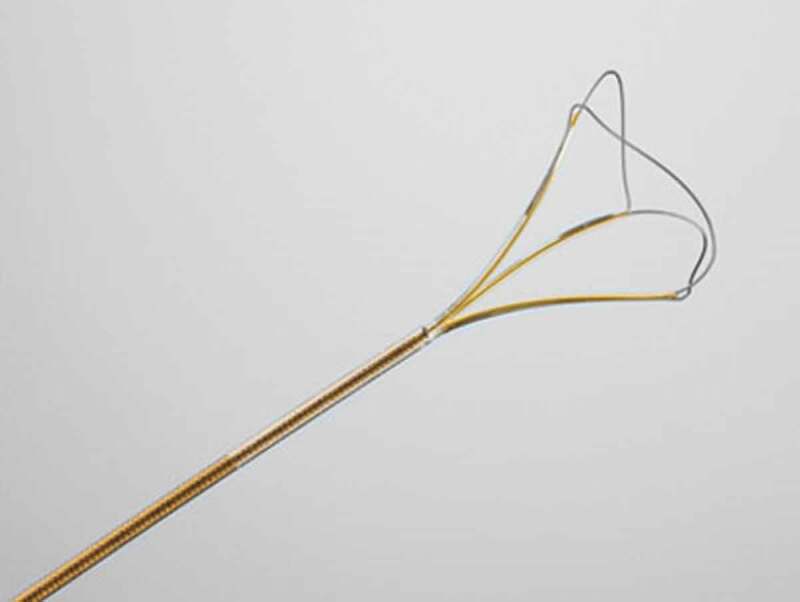
Basket device (N-gage, 1.7 F, Cook Medical, Bloomington, IN, USA) used during the one-surgeon basketing technique

Next, the UAS was introduced over the guidewire and positioned under the ureteral stone or a few centimetres below the pelvis to allow better deflection control of the scope to reduce intrarenal pressure and to facilitate the extraction of large or multiple renal stones. A holmium laser was used to crush the stones to sizes of 2–4 mm by f-URS (Storz FlexX2 or Olympus P6). All stones were managed by laser fragmentation (0.5–1.0 J × 5–10 Hz). In the patients with ureteral stones, 365-μm laser fibres were predominantly used because fibres of this size offer a higher degree of lithotripsy efficiency due to the larger surface area. In the above patients, the patient position was changed to head-down to prevent the stone fragments from moving to the lower pole because the difficulty of retrieving the fragments can be increased in such a scenario. In patients with upper, middle, lower, and renal pelvis calyceal, 200-μm laser fibres were predominantly used because the f-URS flexibility could be deployed maximally. After crushing the observed stones into fragments that could easily be extracted (≤4 mm), a basket catheter (N-gage, 1.7 F; Cook Medical) (Figure 1) with an M-arm (MC Medical) attached to a flexible ureteroscope was inserted.

**Figure 2.**
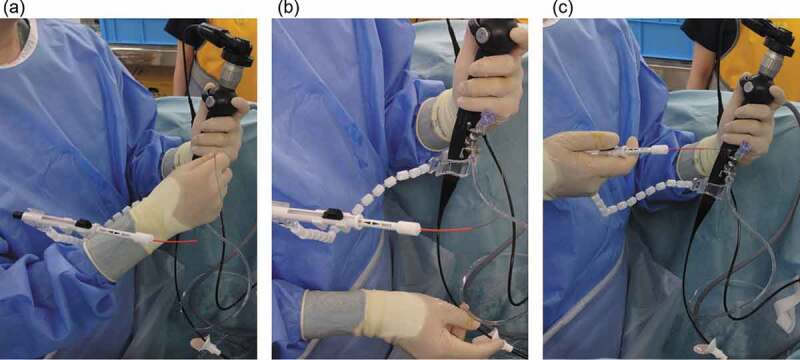
The one-surgeon basketing technique performed during f-URS by a solo surgeon. (a) The basket catheter was inserted after reaching the desired pelvic and renal cup area. (b) The endoscope was inserted so that the basket catheter could reach the target stone. (c) The basket catheter was closed to obtain the target stone

**Figure 3.**
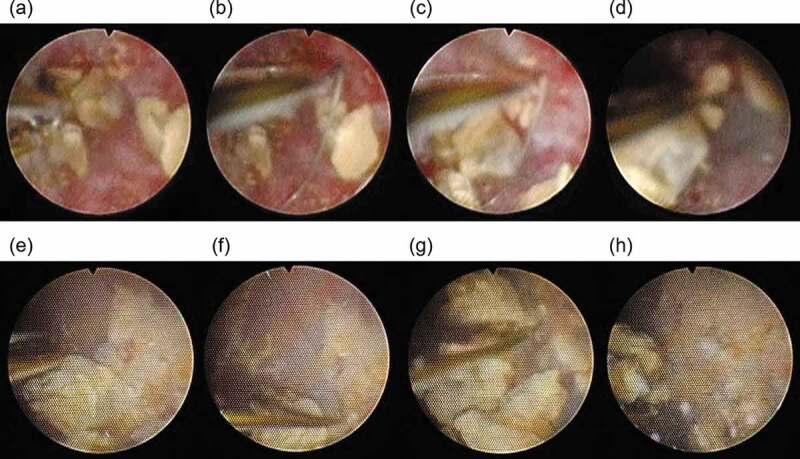
The ‘front catch’ and ‘side catch’ techniques of the one-surgeon basketing technique. (a–d) The front catch technique. (a) A basket catheter was inserted to take up about one-quarter of the screen. (b) The basket catheter was opened to the size of the stone indicated for extraction. (c) The endoscope was advanced straight so that the open basket catheter grasped the stone. (d) The basket catheter was closed to capture the stone. (e–h) The side catch technique. (e) A basket catheter was inserted to take up about one-quarter of the screen. (f)The basket catheter was opened to the size of the stone indicated for extraction. (g) The endoscope was moved left, right, up, and down to get the stone into the open basket. (h) The basket catheter was closed to capture the stone

This basket catheter typically took up about one-quarter of the screen view in front or to the side of the target stone (Figure 2(a)). The flexible ureteroscope was manipulated with the non-dominant hand, and the basket catheter was manipulated with the dominant hand. The basket was half opened, and the flexible ureteroscope moved from the near side to the calculus position (Figure 2(b)). The one-surgeon basketing technique was executed in one of two ways: the front catch (Figure 3(a–d)) or side catch (Figure 3(e–h)) method. In large spaces such as the renal pelvis, a flexible ureteroscope was moved straight ahead to capture stones, while the distance of the basket out of the flexible ureteroscope remained unchanged, which is termed as the ‘front catch’ technique. In contrast, in narrow spaces such as a renal pole, a flexible ureteroscope was moved laterally to capture stones, while the distance of the basket out of the flexible ureteroscope remained unchanged, which is termed as the ‘side catch’ technique. The calculus was confirmed to have entered the basket catheter, and the basket catheter was closed slowly with the dominant hand (Figure 2(c)). The basket catheter holding the calculus was moved a little closer to the flexible ureteroscope, and an endoscope monitor was used to confirm that the calculus had entered the tip of the UAS. The fragments were removed from the body via the UAS. This procedure was repeated by the solo surgeon until all fragments that could be enclosed by the basket catheter had been retrieved. In all patients, a 5-F ureteral stent (Polaris Ultra; Boston Scientific) was placed immediately after f-URS and later removed via flexible cystoscopy with local anaesthesia at 2 weeks after f-URS.

During f-URS, we used the single-action pumping system to provide a high irrigation flow to ensure a clear view was present to help avoid ureteral injury when the flexible ureteroscope was inserted from the UAS into the ureter and renal pelvis. Essentially, the single-action pumping system was not used, except in patients with poor visual access when the stones were being crushed and retrieved. This procedure prevented overpressure within the renal pelvis.

### Parameters

Preoperative clinical information included age, sex, body mass index (BMI), stone number, stone size (mm), stone location (kidney [upper or middle calyceal or lower calyceal or renal pelvis calyceal] or ureter), stone side (right or left), CT attenuation value (HU), impacted stone, and hydronephrosis. An impacted stone was defined as a stone through which a guidewire could not be passed at the initial attempt, and mucosal edema, ureteral polyps, strictures, and stone fixation on the ureter were endoscopically found. In patients with multiple stones, the stone size was calculated as the sum of the diameters of all stones. Perioperative clinical information included total operating time (min), stone fragmentation time (min), stone retrieval time (min), total laser energy (kJ), and complications, such as ureteral injury and macrohaematuria. Postoperative clinical information included SFR, postoperative fever, estimated GFR change (mL/min/1.73 m^2^), haemoglobin change (g/dL), white blood cell count change (10^3^/µL), C-reactive protein change (mg/dL), postoperative hospital stay (h), and postoperative emergency visits. The definition of being stone free was residuals of ≤2 mm at kidney–ureter–bladder (KUB) 1 month after f-URS. Postoperative fever was defined as a significant fever of >38.0°C. The complications were classified according to the modified Clavien–Dindo classification system [12].

## Results

A total of 100 patients were included in this analysis. Patient background details are presented in Table 1. The median patient age was 65 years and 46 patients had kidney stones and 54 had upper ureteral stones. Initial symptoms included 25 patients with pyelonephritis, 34 with pain, 30 with abnormal health evaluation findings, eight with haematuria, and three with acute renal failure. The number of stones per patient ranged from one to three: 64 patients with one, 27 with two, and nine with three stones. The median stone size among our cohort was 14 mm. In all, 40 patients had been implanted with a JJ stent before f-URS, whereas 22 patients presented with impacted stones.

**Table 1.** Patients’ baseline characteristics

Variable	Value
Age, years, median (range)	65 (31–87)
Sex, male, *n* (%)	55 (55)
BMI, kg/m^2^, median (range)	24 (16–34)
Stone number, median (range)	1 (1–3)
Stone size, mm, median (range)	14 (5–29)
Stone location, *n* (%)	
Kidney	46 (46)
Upper or middle calyceal	15 (15)
Lower calyceal	21 (21)
Renal pelvis calyceal	10 (10)
Upper ureter	54 (54)
Right/left	42/58
CT attenuation value, HU, median (range)	1200 (320–2100)
Impacted stone, *n* (%)	22 (22)
Hydronephrosis, *n* (%)	59 (59)
Preoperative ureteral stent, *n* (%)	40 (40)

The patients’ results details are presented in Table 2. The median operative time was 74 min. The median stone fragmentation time was 15 min and the median stone retrieval time was 30 min. The SFR was 91% at 1 month postoperatively. The SFR was 87% for kidney stones and 94% for ureteral stones (*P* = 0.29). During the one-surgeon basketing none of the patients had poor visibility due to haematuria. Also, none of the patients required an assistant during the stone retrieval process. The median length of postoperative stay was 1 day. Four patients had postoperative emergency visits due to stone-related pain (two) and macrohaematuria (two). Complications related to stone retrieval were identified in two patients (2%); two patients had Clavien–Dindo Grade IIIa ureteral injury caused by the basket device. Considering total intraoperative complications, ureteral injury occurred in five patients (5%), with the degree of injury ranging between Clavien–Dindo Grades II and IIIa. Postoperative fever was recorded in five patients (5%). In this study, the stone components were calcium oxalate in 84% of cases, calcium phosphate in 10%, and uric acid in 6%.

**Table 2.** Patients’ perioperative characteristics

Variable	Value
Total operation time, min, median (range)	74 (25–148)
Fragmentation time, min, median (range)	15 (1–87)
Retrieval time, min, median (range)	30 (2–91)
Total energy, kJ, median (range)	2.1 (0.2–26.7)
SFR, *n* (%)	91 (91)
Postoperative hospital stay, days, median (range)	1 (1–5)
Complication, *n* (%)	
Ureteral injury	5 (5)
Postoperative fever	5 (5)
Estimated GFR change, mL/min/1.73 m^2^, median (range)	0.5 (–20 to +25)
Haemoglobin level change, g/dL, median (range)	–0.9 (–3.2 to +1.3)
White blood cell count change, 10^3^/μL, median (range)	+1600 (–5700 to +13,000)
C-reactive protein level change, mg/dL, median (range)	+0.2 (–3.4 to +5.0)

A rigid ureteroscope was inserted into the ureter in all patients, which dilatated the ureter and facilitated easier UAS insertion. In patients with an impacted ureteral stone, the stone was crushed by the rigid ureteroscope and pushed back to the renal pelvis, and then the UAS was placed and f-URS was performed. A 12/14-F UAS and 9.5/11.5-F UAS were used in 95 and five of the 100 patients, respectively. The ureteral dilator was used prior to UAS insertion: 8 F in two and 10 F in 89 patients. Severe ureteral stenosis requiring a ureteral balloon was not observed in the present study.

## Discussion

Generally, use of the basketing retrieval technique necessitates an assistant to operate the basket device. However, the one-surgeon basketing technique in the present study could be performed by just one person; thus, one of the advantages of the one-surgeon basketing technique is the possibility for a solo surgeon to perform f-URS with active retrieval basketing. It has been reported that the one-surgeon basketing technique is comparable to the two-person basketing technique [10]. All patients of f-URS were performed safely in the present study and the applied one-surgeon basketing technique was considered an effective method of stone fragment removal.

Comparing the results reported in relation to the dusting technique and conventional basketing retrieval technique, our present operation time and SFR were comparable with the previous reports [10,13,14] in Table 3. The one-surgeon basketing technique tends to be difficult to perform but, with the right instruction, can be conducted adequately by a new operator within the span of just a few cases [11]. Therefore, the one-surgeon basketing technique is not extremely difficult to learn.

**Table 3.** Comparison of our present results and other reports on basketing retrieval

Reference	Patients, *n*	Age, years	Stone diameter, mm	Operation time, min	One- or two-person technique	SFR, %	Stone-free definition	Laser energy, kJ
Humphreys et al. [13]	82	54	9	67	Two-person	74.3	No residual stone	20.2
Lee et al. [14]	172	56	11	83	Two-person	89	≤3 mm	NA
Tabei et al. [10]	109	64	10	85	Two-person	61.5	No residual stone	NA
Tabei et al. [10]	87	65	13	80	One-person	90.8	No residual stone	NA
Present series	100	65	14	74	One-person	91	≤2 mm	2.1

Data are shown as sample median or mean. NA: not applicable.

The advantages of performing basketing retrieval as compared with dusting encompass several points. One of the advantages is the ability to extract the complete stone in uncomplicated cases, and calculus analysis is possible from stone fragmentation. Second, the total laser energy is typically lower with basketing retrieval than with the dusting technique [15,16]. Low laser energy may result in lowering the urinary tract temperature, resulting in less urinary heat damage [15,16]. However, given the use of UAS is essential in the basketing retrieval approach, such use might injure the ureter in some cases [17]. Overall, one cannot clearly state that either the basketing retrieval technique or dusting technique is better; instead, each technique has its unique advantages and disadvantages, and surgeons should be familiar with both techniques and choose between them on a case-by-case basis [18]. It has been reported that about two-thirds of urologists use the dusting technique during f-URS [3]. However, it is necessary to decide which method is appropriate with consideration of the patient background, stone size, and stone position. In patients with infected stones, poor performance status, or with single kidneys, complete removal of stones might be considered as particularly useful for preventing postoperative complications. If the one-surgeon basketing technique is acquired, basketing retrieval could be possible in various cases, such as in those mentioned above.

At community hospitals with one or two urologists, f-URS can be done using the one-surgeon basketing technique by a single surgeon, which can be considered a great advantage. One of the important tools in the one-surgeon basketing technique is the basket holder. With the basket holder, when the flexible ureteroscope was manipulated with the non-dominant hand, the basket catheter was manipulated with the dominant hand. In the one-surgeon basketing technique, there are two catch methods, that is, front catch and side catch. In a large space, opening the basket well in front of the stone and advancing the endoscope to capture the stone into the open basket from the front is feasible. Conversely, in a smaller space, opening the basket well at the side of the stone and shaking the endoscope to capture the stone from the side into the open basket is likely preferable. Both approaches are effective methods in the one-surgeon basketing technique.

To best of our knowledge, stone fragmentation and stone retrieval times are rarely mentioned in URS papers. In the present study, the median stone fragmentation time and stone retrieval time was 15 and 30 min, respectively. Of course, the time of fragmentation and retrieval depends on the stone size and position. If stones are fragmented into larger pieces, the fragmentation time is reduced. However, the larger pieces of fragmented stone might get stuck in the sheath during retrieval and have to be crushed again. If the stones are crushed into smaller pieces, the number of pieces of fragmented stone increases and the retrieval time might be longer. In the present study, the ratio of fragmentation time to retrieval time was 1:2. Further study will be needed to calculate proper fragmentation and retrieval times according to the stone characteristics.

There are several limitations to the present study. First, this was a retrospective study over a relatively long time; therefore, selection bias could not be ruled out completely. Therefore, a future prospective study conducted under strict conditions is needed to prove the utility of the one-surgeon basketing technique. Second, the present study did not compare the one-surgeon basketing technique with the conventional two-person basketing technique. However, we believe that the one-surgeon basketing technique might be one of the effective surgical methods for f-URS because there was no significant difference in either intra- or postoperative complications as compared with in other reports. We would like to propose that the one-surgeon basketing technique is a useful technique.

## Conclusions

This examination of 100 patients suggests our one-surgeon basketing method is a safe and effective method for stone fragment extraction. The present results also serve as a reference for institutions planning to introduce the one-surgeon basketing technique for stone extraction during f-URS.

## Data Availability

All the data supporting our findings are contained within the manuscript; any missing details will be shared upon request.
